# Which fMRI clustering gives good brain parcellations?

**DOI:** 10.3389/fnins.2014.00167

**Published:** 2014-07-01

**Authors:** Bertrand Thirion, Gaël Varoquaux, Elvis Dohmatob, Jean-Baptiste Poline

**Affiliations:** ^1^Parietal Project-Team, Institut National de Recherche en Informatique et AutomatiquePalaiseau, France; ^2^Commissariat à l’énergie Atomique et Aux Énergies Alternatives, DSV, Neurospin, I2 BMGif-sur-Yvette, France; ^3^Henry H. Wheeler Jr. Brain Imaging Center, University of California at BerkeleyBerkeley, CA, USA

**Keywords:** functional neuroimaging, brain atlas, clustering, model selection, cross-validation, group studies

## Abstract

Analysis and interpretation of neuroimaging data often require one to divide the brain into a number of regions, or parcels, with homogeneous characteristics, be these regions defined in the brain volume or on the cortical surface. While predefined brain atlases do not adapt to the signal in the individual subject images, parcellation approaches use brain activity (e.g., found in some functional contrasts of interest) and clustering techniques to define regions with some degree of signal homogeneity. In this work, we address the question of which clustering technique is appropriate and how to optimize the corresponding model. We use two principled criteria: goodness of fit (accuracy), and reproducibility of the parcellation across bootstrap samples. We study these criteria on both simulated and two task-based functional Magnetic Resonance Imaging datasets for the Ward, spectral and k-means clustering algorithms. We show that in general Ward’s clustering performs better than alternative methods with regard to reproducibility and accuracy and that the two criteria diverge regarding the preferred models (reproducibility leading to more conservative solutions), thus deferring the practical decision to a higher level alternative, namely the choice of a trade-off between accuracy and stability.

## 1. Introduction

Brain parcellations divide the brain’s spatial domain into a set of non-overlapping regions or modules that show some homogeneity with respect to information provided by one or several image modalities, such as cyto-architecture, anatomical connectivity, functional connectivity, or task-related activation. Brain parcellations are therefore often derived from specific clustering algorithms applied to brain images. Such approaches are generally useful because the voxel sampling grid of the reference space, e.g., the MNI template, is most often at a higher resolution than the brain structures of interest, or at a scale that is too fine for the problem under investigation, yielding an excessive number of brain locations and correlated data. In other words, the structures of interest are rarely at the level of a specific voxel, but at the level of many voxels constituting a (possibly small) brain region. Three strategies are commonly used to study function beyond the voxel description: (1) the use of anatomical or functional regions of interest (ROIs), (2) the use of a brain atlas, or (3) the use of data-driven parcellations.

*ROI-based analysis* has been advocated as a way to focus data analysis on some structures of interest and consists in building a summary of the signal in a predefined region (Nieto-Castanon et al., 2003). The choice of the region(s) can be based on prior experiments (e.g., Saxe et al., 2006). Note that in extreme cases, the region can reduce to a single voxel, one reported in previous literature as the peak coordinate of a contrast image^1^. The obvious limitation of ROI-based analysis is that the signal present outside the region under consideration is ignored *a priori*; as a consequence, the results depend heavily on the choice of this ROI, which may not fit well the new data. In the hypothesis testing framework, the smaller number of tests performed may, however, increase the power of the analysis.

*Brain atlases* come into play to provide a set of ROIs that cover the brain volume (among many others see e.g., Mazziotta et al., 2001; Tzourio-Mazoyer et al., 2002; Shattuck et al., 2008). An atlas generally accounts for a certain state of the knowledge of the brain structures (anatomically, functionally or based on connectivity), from which well-defined entities can be distinguished. In other words, an atlas represents a certain *labeling* of brain structures. Often this labeling is linked to an ontology representing the current knowledge (Eickhoff et al., 2011; Cieslik et al., 2012). In spite of their obvious usefulness, existing atlases are limited in two regards: (1) There exist currently many different atlases, but they are mutually inconsistent (Bohland et al., 2009); (2) A given atlas may not fit the data well. Atlas misfits can be due to image characteristics and processing strategies that have evolved since the atlas creation, or because a given study deals with a population that is not well represented by the subjects used to construct the atlas, or because the information of interest is simply not mapped properly in the given atlas. Atlas misfit is often pronounced with regards to mapping brain function; for instance most anatomical atlases have large frontal brain regions that many researchers would rather divide into smaller ones with more precise functional roles.

Unlike brain atlases, also used to define regions of interest, brain parcellations are data-driven. They do not reflect a pre-defined ontology of brain structures—known anatomical names and concepts—but they may much better represent the measurements or features of interest, i.e., they provide a better model of the signal (Flandin et al., 2002; Simon et al., 2004; Thirion et al., 2006; Lashkari et al., 2010, 2012). The (anatomical) labeling of these parcels can then be performed with the most appropriate atlas.

While functional parcellations can be used in different contexts, we focus here on finding a well-suited model to obtain local averages of the signal for group studies. These parcel averages can be thought of as a data reduction adapted to various tasks, such as the estimation of brain-level connectivity models (see e.g., Yeo et al., 2011; Craddock et al., 2012), of physiological parameters (Chaari et al., 2012), for group analysis (Thirion et al., 2006), the comparison of multiple modalities (Eickhoff et al., 2011) or in multivariate models (Michel et al., 2012). This is especially useful for the analysis of large cohorts of subjects, because this step can reduce the data dimensionality by several orders of magnitude while retaining most of the information of interest. We will show in this paper that common brain atlases, merely reflecting sulco-gyral anatomy, are not detailed enough to yield *adequate* models of the (functional) data.

Data-driven parcellations can be derived from various image modalities reflecting different neurobiological information, for instance T1 images with anatomical information, such as gyro-sulcal anatomy (Desikan et al., 2006; Klein and Tourville, 2012), post-mortem *in vitro* receptor autoradiography for cyto-architecture (Eickhoff et al., 2008; Fischl et al., 2008), anatomical connectivity (Roca et al., 2010) with diffusion imaging, or functional features with BOLD data. In this work, we focus on the latter, that we call *functional parcellations*. These parcellations are currently derived either from resting-state functional Magnetic Resonance Images (rs-fMRIs) (Yeo et al., 2011; Blumensath et al., 2012; Craddock et al., 2012; Kahnt et al., 2012; Wig et al., 2013), from activation data (Flandin et al., 2002; Lashkari et al., 2010, 2012; Michel et al., 2012), or from meta-analyses (Eickhoff et al., 2011). To investigate which parcellations are most appropriate, we restrict our work to activation data that have more tractable probabilistic models than resting-state data. We also omit edge-based parcellation methods, such as those described in Wig et al. (2013) or Cohen et al. (2008): while these are certainly useful to segment the cortical surface by revealing abrupt changes in the functional connectivity patterns when crossing region boundaries, they do not lend themselves to model selection due to the absence of a probabilistic framework. This family of approaches is certainly an interesting competitor for future analyzes of functional parcellations performed on the cortical surface.

The most popular parcellation techniques are mixture models (Golland et al., 2007; Tucholka et al., 2008; Lashkari et al., 2010, 2012), variants of the k-means algorithm (Flandin et al., 2002; Yeo et al., 2011; Kahnt et al., 2012), hierarchical clustering (Eickhoff et al., 2011; Michel et al., 2012; Orban et al., 2014) and variants thereof (Blumensath et al., 2012), spectral clustering (Thirion et al., 2006; Chen et al., 2012; Craddock et al., 2012) and dense clustering (Hanson et al., 2007). Some of these approaches, but not all, impose spatial constraints on the model, and therefore provide spatially connected spatial components. In the multi-subject setting, some models adapt the spatial configuration to each subject (e.g., Thirion et al., 2006; Lashkari et al., 2010, 2012), but most approaches do not. Parcellations can also be obtained from dictionary learning techniques such as independent components analysis (ICA) and variants of principal components analysis (PCA) (Kiviniemi et al., 2009; Varoquaux et al., 2010, 2011, 2013; Abraham et al., 2013). These rely on a linear mixing approach that changes the nature of the problem and implies other probabilistic models.

While parcellation techniques have great potential and can serve as the basis of many further analyses, it is important to assess their relative performance. To the best of our knowledge, no systematic comparison of parcellation methods has been carried out in previous work.

The comparison between clustering techniques is only relevant if for each technique the best possible model is selected. It turns out that model selection for clustering is a notoriously difficult problem, as is any unsupervised problem in which one wishes to identify some structure in noisy data. While in practice the choice of the model may depend on the context of the study [for instance, fitting a given target of interest using region-based signal averages Ghosh et al. (2013)], here we derive general rules to compare parcellation models from empirical observations. In the context of brain mapping, two criteria are particularly relevant for model selection: (1) the *goodness of fit* or *accuracy* of a model, i.e., the ability of the parcellation extracted to model properly the signals of interest on observed and unobserved data, and (2) *stability*, i.e., the consistency of the parcellations obtained from different sub-groups of a homogeneous population. Importantly, there is *a priori* no reason why these two criteria should give consistent answers. There have been few attempts to tackle this, such as Tucholka et al. (2008), Kahnt et al. (2012), and Ghosh et al. (2013), but these approaches did not model the multi-subject nature of the signal; moreover (Tucholka et al., 2008) were subdividing prior gyrus definition (hence not brain-wide) and they did not benchmark different clustering techniques. In the present work, we present experiments on simulated and real data using different clustering techniques and proper accuracy and reproducibility criteria. To make this tractable computationally and to obtain clear interpretation, we limit ourselves to non-linear mixing models, i.e., clustering approaches. Note that methods comparison for clustering versus linear mixing models (ICA, variants of sparse PCA) has been addressed e.g., in Abraham et al. (2013), while model order selection for linear model-based region extraction is still an open problem. For similar reasons, we consider the case in which parcels are identical for all subjects.

The remainder of the paper is organized as follows: in section 2, we introduce the methods tested in this work and the criteria for model evaluation; in section 3 we describe our experiments on simulated and real data, the results of which are given in section 4. Conclusions on the choice of optimal processing algorithms and the selection of parcellation schemes are drawn in section 5.

## 2. Materials and methods

### 2.1. Notation

We start with a given set of *n* functional images that represent e.g., different contrasts in a given group of subjects. We denote *N* to be the number of subjects and *F* the number of functional images (here contrasts) per subject, such that *n* = *NF*. These images are typically the results of first-level analysis (standardized effects) and are sampled on a grid of *Q* voxels. Starting from *n* fMRI volumes **Y** = [**y**^1^, …, **y**^*Q*^] ∈ ℝ^*n* × *Q*^ that consist of *Q* voxels, we seek to cluster these voxels so as to produce a reduced representation of **Y**.

### 2.2. Clustering methods for brain functional parcellation

#### 2.2.1. K-means algorithm

K-means is arguably the most used clustering technique for vector data. It consists of an alternate optimization of (1) the assignment *u*_k−means_ of samples to cluster and (2) the estimation of the cluster centroids.

(1) $$\forall j\in [1,Q],\;u_{\text{k-means}}(j)={\text{argmin}}_{c\in \;[1,\ldots ,K]}\left\|{\langle Y\rangle }_{c}-y^{\;j}\right\|$$

(2) $${\langle Y\rangle }_{c}≜\frac{1}{\left|c\right|}\sum_{u_{\text{k-means}}(\;j)\;=\;c}y^{\;j}$$

It explicitly minimizes the inertia, i.e., the sum of squared differences between the samples and their representative cluster centroid. We introduce an approximation for the sake of efficiency: the whole set of feature data used in clustering (several contrasts from all the subjects) of dimension *n* = *N(subjects)* × *F (contrasts)* is reduced by PCA to *m* = 100 components prior to clustering, capturing about 50% of the variance. It is important to note that k-means clustering of fMRI data are used without explicitly considering their spatial structure, although spatial smoothing prior to clustering can indirectly provide spatial regularization.

#### 2.2.2. Ward’s algorithm

As an alternative, we consider a *hierarchical agglomerative clustering* (Johnson, 1967). These procedures start with every voxels **x**^*j*^ representing singleton clusters {*j*} and, at each iteration, a pair of clusters, selected according to a criterion discussed below, is merged into a single cluster. This procedure yields a hierarchy of clusters represented as a binary tree 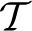, also often called a dendrogram (Johnson, 1967), where each non-terminal node is associated with the cluster obtained by merging its two children clusters.

Among different hierarchical agglomerative clustering procedures, we use the variance-minimizing approach of Ward’s algorithm (Ward, 1963). In short, two clusters are merged if the resulting cluster minimizes the sum of squared differences of the fMRI signal within all clusters. More formally, at each step of the procedure, we merge the clusters *c*_1_ and *c*_2_ that minimize

(3) $$\begin{array}{l} \Delta (c_{1},c_{2})=\sum_{j\;\in \;c_{1}\;\cup \;c_{2}}{\left\|y^{\;j}-{\langle Y\rangle }_{c_{1}\;\cup \;c_{2}}\right\|}_{2}^{2}\\ \;-\left(\sum_{j\;\in \;c_{1}}{\left\|y^{\;j}-{\langle Y\rangle }_{c_{1}}\right\|}_{2}^{2}+\sum_{k\;\in \;c_{2}}{\left\|y^{\;k}-{\langle Y\rangle }_{c_{2}}\right\|}_{2}^{2}\right)\\ \;=\frac{\left|c_{1}||c_{2}\right|}{\left|c_{1}|+|c_{2}\right|}{\left\|{\langle Y\rangle }_{c_{1}}-{\langle Y\rangle }_{c_{2}}\right\|}_{2}^{2}, \end{array}$$

where 〈**Y**〉_*c*_ is the average vector defined in Equation (2). In order to take into account the spatial information, we also add connectivity constraints in the hierarchical clustering algorithm, so that only neighboring clusters can be merged together. In other words, we try to minimize the criterion Δ(*c*_1_, *c*_2_) only for pairs of clusters that share neighboring voxels. Given a number of parcels *K*, we stop the construction of the tree at the (*Q* − *K*)th iteration and retain the corresponding assignment *u*_ward_. Note that the data are subject to the same PCA procedure as for k-means clustering.

#### 2.2.3. Spectral clustering

Spectral clustering (Shi and Malik, 2000; Ng et al., 2001) consists in performing k-means clustering on a representation of the data that preserves the spatial structure yet represents the functional features’ similarity^2^. This representation is typically obtained by using the first eigenvectors of the Laplacian matrix of the graph that encodes the spatial relationships weighted by the functional features similarity between adjacent locations. For all voxel pairs (*i*, *j*) ∈ [1 …, *Q*]^2^, Let

(4) $$W_{ij}=\{\begin{array}{l} \exp\text{​}\left(-\frac{{\left\|y^{\;i}-y^{\;j})\right\|}^{2}}{2\sigma_{f}^{2}}\right)\text{ if i and j are neighbors}\\ 0\text{ otherwise} \end{array}$$

where we used σ^2^_*f*_ = mean_*i*~*j*_‖**y**^*i*^ − **y**^*j*^‖^2^, where the averaging is performed over all pairs of adjacent voxels. σ^2^_*f*_ is thus the average squared distance between the data across neighboring voxels. *W* is therefore an adjacency matrix weighted by the functional distance between voxels. We denote Δ_*W*_ the diagonal matrix that contains the sum of the rows of *W*.

Then, let (ξ_1_, …, ξ_*m*_) the first *m* solutions of *W*ξ = λΔ_*W*_ξ. The spectral clustering of the dataset is defined as:

(5) $$u_{\text{spectral}}=\text{k-means}\left(\left[\xi_{1},\ldots ,\xi_{m}\right]\right),$$

*m* = 100 in our experiments. We also tried different (larger or smaller) values, but those did not yield significantly better solutions.

#### 2.2.4. Geometric clustering

To provide a reference for comparison, we also use a clustering algorithm that does not take into account the functional data, but only the spatial coordinates of the voxels. In practice, it is obtained through a k-means clustering of the spatial coordinates.

### 2.3. A mixed-effects model of the signal within parcels

We introduce a probabilistic model of the signal of the voxels in a given (fixed) parcel 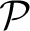
_*k*_, *k* ∈ [1, …, *K*], that includes a random subject effect. Let us first assume that we work with one functional image (*F* = 1). Let *p* be the number of voxels in the parcel, pooled across subjects: it is the size of the parcel multiplied by *N*; let **y** be a *p*−dimensional vector that denotes the scalar signal in the voxels contained in 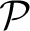
_*k*_, concatenated across subjects; we model it though the following mixed-effects model:

(6) y=μ1+Xβ+ε,

where μ is the average signal within the parcel, **1** is a vector of ones of length *p*, β is a vector of subject-specific random effects parameters, **X** the (known) matrix that maps subjects to voxels: for each row, a one in the *i*th column indicates that the value is from subject *i*. ε represents the intra-subject variability of the signal within a parcel. It is further assumed that ε and β are independent, normal and centered at 0, with variance σ^2^_1_ and σ^2^_2_ that express respectively the within and between subject variance. The probabilistic model of **y** is thus:

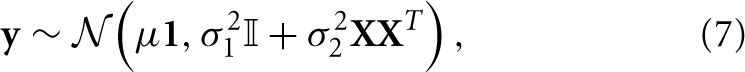



where 𝕀 is the *p* × *p* identity matrix.

The generalization to non-scalar images (for instance, *F* > 1 images per subject) is obtained by assuming the independence of the observations conditional to the parcellation, hence it decouples into multiple (*F*) scalar models. The estimation of the parameters (μ, σ_1_, σ_2_) is carried out in each parcel 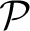
_*k*_, *k* ∈ [1, …, *K*] using the maximum likelihood principle; we use an Expectation-Maximization algorithm to estimate the model parameters (Meng and van Dyk, 1998).

### 2.4. Model selection for functional parcellations

A problem that comes naturally with clustering algorithms is the choice of the number *K* of clusters to be used in the model. To guide this choice we consider four standard measures: BIC, cross-validated likelihood, adjusted rand index, and normalized mutual information.

#### 2.4.1. Bayesian information criterion, BIC

The goodness of fit of a probabilistic model is given by the log-likelihood of the data and the quality of the model is easily measured using the BIC criterion (Schwarz, 1978), that penalizes the negative log-likelihood by the number of parameters used. Within a given parcel 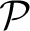
_*k*_, this yields the following:

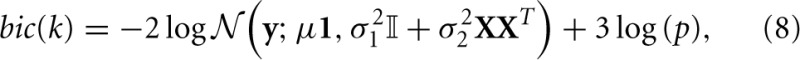



Where 3 is the number of parameters of the model (μ, σ_1_, σ_2_). Note that all the quantities in this formula (**y**, μ, σ^2^_1_, σ^2^_2_, **X**, *p*) depend on *k*, the index of the parcel. *bic*(*k*) is summed across parcels in order to yield a unique quantity that is comparable for different values of *K*, that we denote *BIC* henceforth.

The BIC is theoretically asymptotically optimal for model selection purpose (Schwarz, 1978), however, it may fail in practice for several reasons. In particular, it relies on some hypotheses for the data, such as the i.i.d structure of the residuals, which are violated in fMRI. This means that the goodness of fit of over-parameterized models increases faster than it should in theory, and thus that more complex models, i.e., with a large number of parcels, are systematically and spuriously preferred. In the case of brain volume parcellation, the violation of the i.i.d. hypothesis might be related to different factors, such as data smoothness or spatial jitter across individuals.

#### 2.4.2. Cross-validated likelihood

A nice feature of the model (Equation 7) is that it can be evaluated on test data, thus making it possible to run a cross-validation procedure on different subjects; such a procedure does not *overfit*, where overfit means *models non-reproducible noise, creating the optimistic bias inherent when learning and evaluating a model on the same data*. We use the log-likelihood in a *shuffle-split* cross-validation scheme: for each fold, the model is learned on the training set (i.e., a random subsample of 80% of the data): this includes the estimation of the clustering and fitting the mixed-effects model; the log-likelihood computed on the test data is then summed across parcels in order to yield a unique quantity, denoted *CV* − *LL* in the following.

#### 2.4.3. Reproducibility by bootstrap

The two previous metrics only address the fit of the data by the model. Another important criterion in neuroimaging is reproducibility (LaConte et al., 2003), which we define in this context as the consistency of two clustering solutions across repeats on bootstrap samples taken from the data, measured by assignment statistics of voxels to clusters. To estimate reproducibility, we repeated the clustering by bootstrapping over subjects and assessed the stability of the clustering between pairs of bootstrap samples using two standard metrics: adjusted mutual information or adjusted rand index. The **adjusted Rand index** (ARI) is comprised between −1 and 1, and measures the consistency of the two labelings while being invariant to a permutation of the labels (Vinh et al., 2009). A value of 1 means perfect correspondence of the labeling, while a value of 0 implies that the correspondence are at chance. An important feature of the ARI metric is that it scales well when the number of clusters *K* is large. See http://en.wikipedia.org/wiki/Rand_index for more details.

**Adjusted mutual information** (AMI) upper bounded by 1, and possibly negative, is an estimate of the mutual information of two discrete assignment of voxels to parcels, which is corrected for chance: two statistically independent assignments should have an AMI value of 0, while two identical assignments should have an AMI value of 1 http://en.wikipedia.org/wiki/Adjusted_mutual_information.

### 2.5. Implementation

We use the algorithms and metrics from the scikit-learn toolbox (Pedregosa et al., 2011). In particular, Ward’s algorithm is very efficient on data size of typical brain images. The following version of the software were used:Matlab R2013A, version 8.1.0.64, SPM8 v. 5242, scikit learn v. 0.14. The code used in this work is available at https://github.com/bthirion/frontiers_2014.

## 3. Experiments

### 3.1. Simulated data

**Figure 1:**
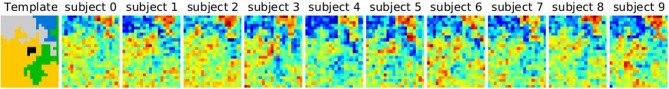
**Example of simulated data used in the 2D simulation experiment**. The *template* or ground truth labeling is shown on the left side, and 10 individual datasets are sampled according to the model, jittered spatially by 2 pixels and then smoothed with a kernel of fwhm 1.17 pixels.

Data are simulated according to model (Equation 6): on a 2D grid of shape 20 × 25 pixels, five random clusters are generated with a hierarchical clustering approach, by using Ward’s parcellation on a set of random signals; 10 individual datasets are sampled using the generative model: for each parcel the parameters μ are sampled from 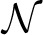
(0, 1), σ_1_ = 1 and the random subject effect β are drawn from 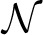
(0, 1), σ_2_ = 1. Note that the βs are kept constant across parcels. Data corresponding to a sample of 10 subjects are generated. To make the data more realistic, we add a deformation to each individual dataset that has a magnitude of 0, 1, or 2 pixels in each direction and smooth it—or not—with a kernel of full width at half maximum (fwhm) of 1.17 pixel. Note, however, that this breaks (on purpose) the hypotheses of the generative model and makes the simulations more realistic. An example is shown in Figure 1.

The question that we address is whether we can hope to recover the true number of clusters from the simulation; to do so, we can use one of the three selection criteria: BIC, cross-validation and bootstrap reproducibility (we use B-AMI by default, but B-ARI yields similar results on this dataset). The recovery is quantified through the adjusted rand index between the true labeling of voxels and the obtained one. The results are based on 200 replications of the experiment, and the optimal number *K* of parcels is searched in the {2, 3, 4, 5, 6, 7, 8, 9, 10, 15, 20, 30} set.

### 3.2. Functional localizer data

Data were acquired from 128 subjects who performed a *functional localizer* protocol as described in Pinel et al. (2007) and referred to as *Localizer* henceforth. This protocol is intended to activate multiple brain regions in a relatively short time (128 brain volumes acquired in 5 min) with 10 experimental conditions, allowing the computation of many different functional contrasts: left and right button presses after auditory or visual instruction, mental computation after auditory or visual instruction, sentence listening or reading, passive viewing of horizontal and vertical checkerboards. The subjects gave informed consent and the protocol was approved by the local ethics committee.

In 59 of the subjects, functional images were acquired on an 3T Siemens Trio scanner using an EPI sequence (TR = 2400 ms, TE = 60 ms, matrix size = 64 × 64, FOV = 19.2 cm × 19.2 cm). Each volume consisted of 40 3 mm-thick axial slices without gap. A session comprised 132 EPI scans, of which the first four were discarded to allow the MR signal to reach steady state. The slices were acquired in interleaved ascending order. Anatomical fSPGR T1-weighted images were acquired on the same scanner, with a slice thickness of 1.0 mm, a field of view of 24 cm and an acquisition matrix of 256 × 256 × 128 voxels, resulting in 124 contiguous double-echo slices with voxel dimensions of (1.0 × 1.0 × 1.0) mm^3^.

In 69 of the subjects, functional and anatomical images were acquired on a 3T Bruker scanner. Functional images were acquired using an EPI sequence (TR = 2400 ms, TE = 60 ms, matrix size = 64 × 64, FOV = 19.2 cm × 19.2 cm). Each volume consisted of *n_a_* 3-mm- or 4-mm-thick axial slices without gap, where *n_a_* varied from 26 to 40 according to the session. A session comprised 130 scans. The first four functional scans were discarded to allow the MR signal to reach steady state. Anatomical T1 images were acquired on the same scanner, with a spatial resolution of (1.0 × 1.0 × 1.2) mm^3^.

The data were subject to a pre-processing procedure that includes the correction of the difference in slice timing, motion estimation and correction, co-registration of the EPI volumes to the T1 image, non-linear spatial normalization of T1 images, then of the fMRI scans to the SPM T1 template. All of these steps were performed using the SPM8 software. Optionally, we considered a 5 mm isotropic smoothing the normalized images. In parallel, an average mask of the gray matter was obtained from the individual normalized anatomies, subsampled at the fMRI resolution, and used to mask the volume of interest in the functional dataset. This procedure yields approximately *Q* = 57,000 voxels at 3 mm resolution.

A General Linear Model (GLM) analysis was applied for the volume using the Nipy package http://nipy.sourceforge.net/. The model included the 10 conditions of the experiments convolved with a standard hemodynamic filter and its time derivative, a high-pass filter (cutoff:128s); the procedure included an estimation of the noise auto-correlation using an AR(1) model.

Activation maps were derived for six functional contrasts, that display the activations related to *left versus right button presses*, *motor versus non-motor tasks*, *sentence listening versus sentence reading*, *computation versus sentence reading*, *reading versus passive checkerboard viewing*, *vertical versus horizontal checkerboard viewing*. We consider that these six contrasts give the most usable summary of the topographic information conveyed by the initial 10 conditions, without obvious redundancies, while avoiding non-specific effects.

The standardized effects related to these *F* = 6 contrasts are used for parcellation fit and evaluation. We consider the possible range of values for *K*: 10, 20, 30, 40, 50, 70, 100, 150, 200, 300, 400, 500, 700, 1000, 1500, 2000, 3000, 5000, 7000, 10000. We consider the value of the different criteria for different *K* values.

### 3.3. HCP data

A set of *N* = 67 subjects of the Human Connectome Project (HCP) dataset was also used in our experiments. These subjects are part of the Q2 release; we used the task-fMRI dataset, that comprises seven different sessions (see Barch et al., 2013 for details), all of which are used here. Starting from the preprocessed volume data provided by the HCP consortium, these dataset were analyzed similarly to the *Localizer* dataset, using the Nipy software for the GLM analysis, that was carried out using the paradigm information provided with the data. The same gray matter mask was used as for the Localizer dataset was used to facilitate comparisons between the two datasets.

In order to reduce computation time, a subset of *F* = 9 functional contrasts were used: the *faces-shape* contrast of the *emotional* protocol, the *punish-reward* contrast of the *gambling* protocol, the *math-story* contrast of the *language* protocol, the *left foot-average* and *left hand-average* contrasts of the *motor* protocol, the *match-relation* contrast of the *relational* protocol, the *theory of mind-random* contrast of the *social* protocol and the *two back-zero back* contrast of the *working memory* protocol. this choice was meant to sample a significant set of cognitive dimensions tested in the protocol, without being exhaustive.

## 4. Results

### 4.1. Simulations

**Figure 2:**
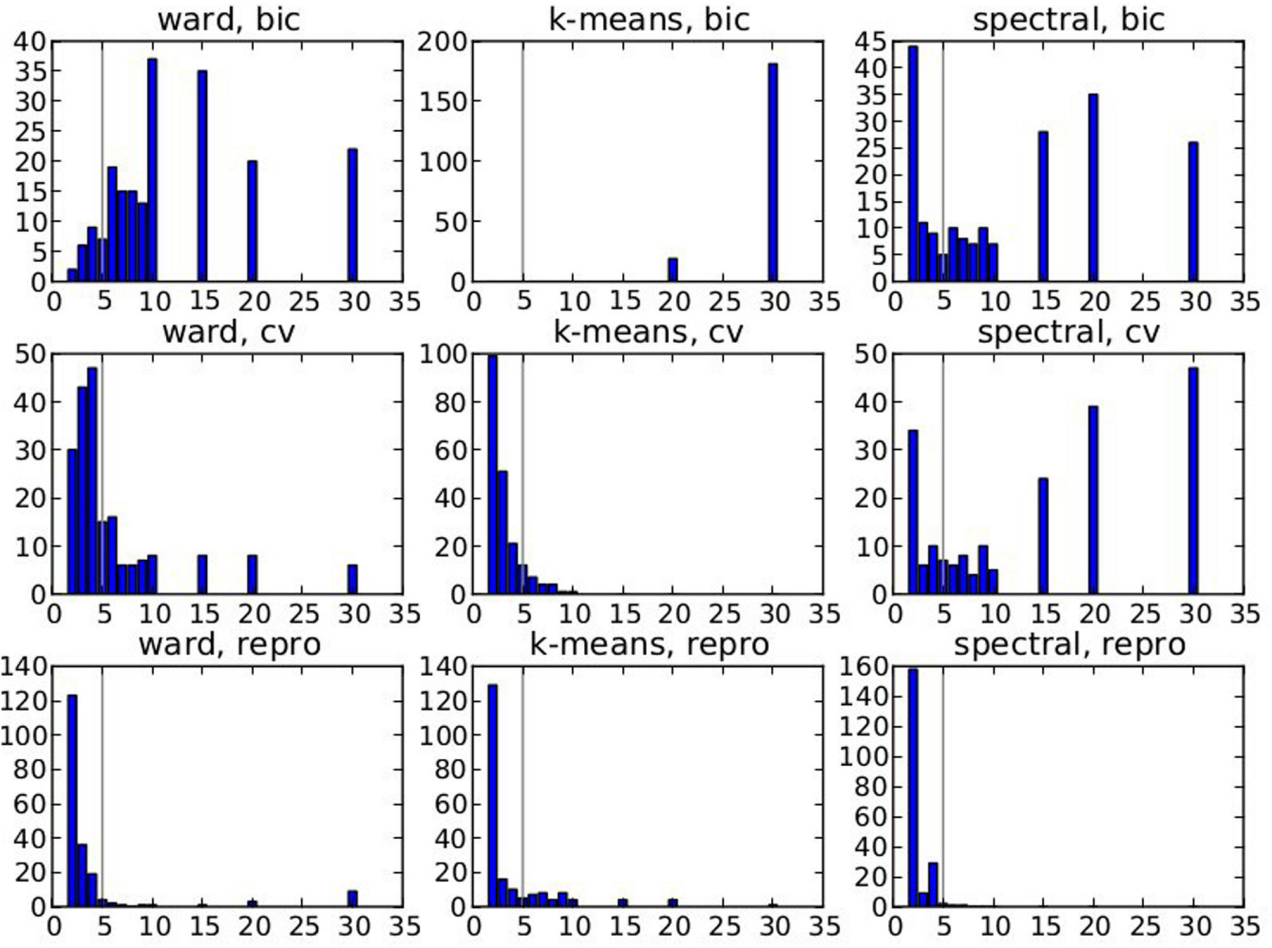
**Results of the simulations: choice of the number parcels for different clustering methods and cluster selection techniques**. Note that the range of possible values is [2, 3, 4, 5, 6, 7, 8, 9, 10, 15, 20, 30] and that the true value is 5. The results are based on data smoothed with a kernel of size 0.5 voxel, and under spatial jitter of 1 voxel isotropic, and are presented across 200 replications. bic, cv (cross validation) and repro (Adjusted Mutual Information) represent three model selection approaches, while ward, k-means and spectral represent three different clustering approaches.

Figure 2 displays the selected K^*^ value, based on data smoothed with a kernel of size 0.5 voxel, and under spatial jitter of 1 voxel isotropic; given that *K^true^* = 5 it shows that BIC tends to select too large number of clusters, while, on the opposite, reproducibility, measured via bootstrapped AMI, is conservative; cross-validated log-likelihood shows an intermediate behavior, as it is conservative for spectral clustering and anti-conservative for k-means. However, the right model is not recovered in general, because the true clustering is not in the solution path of the different methods (this is especially true for spectral clustering), or because model selection fails to recover the right number of parcels.

Our main observation is thus that reproducibility-based model selection criteria seem over-conservative, while accuracy-based selection criteria are too liberal.

### 4.2. Real data

#### 4.2.1. Qualitative assessment of the solutions

**Figure 3:**
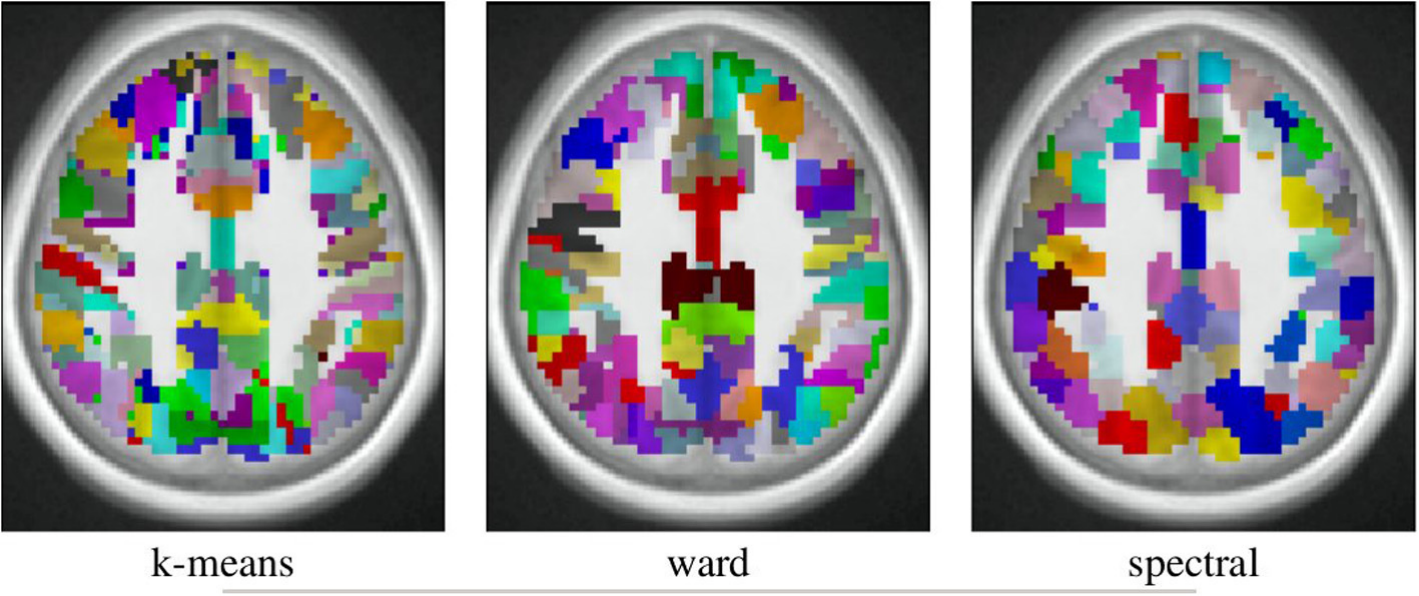
**Example of parcellation with 500 parcels on the Localizer dataset**.

The spatial layout of the clusters can be observed in the brain volume (see Figure 3 for on axial slice), and it represents the characteristics of the competing clustering algorithms: Spectral clustering yields a very geometrical parcellation of the volume, hinting at a lower sensitivity to the functional input data, while k-means presents results with less spatial consistency (e.g., disconnected clusters), yet a realistic representation of plausible functional patches, and Ward’s algorithm presents a compromise between the two solutions.

**Figure 4:**
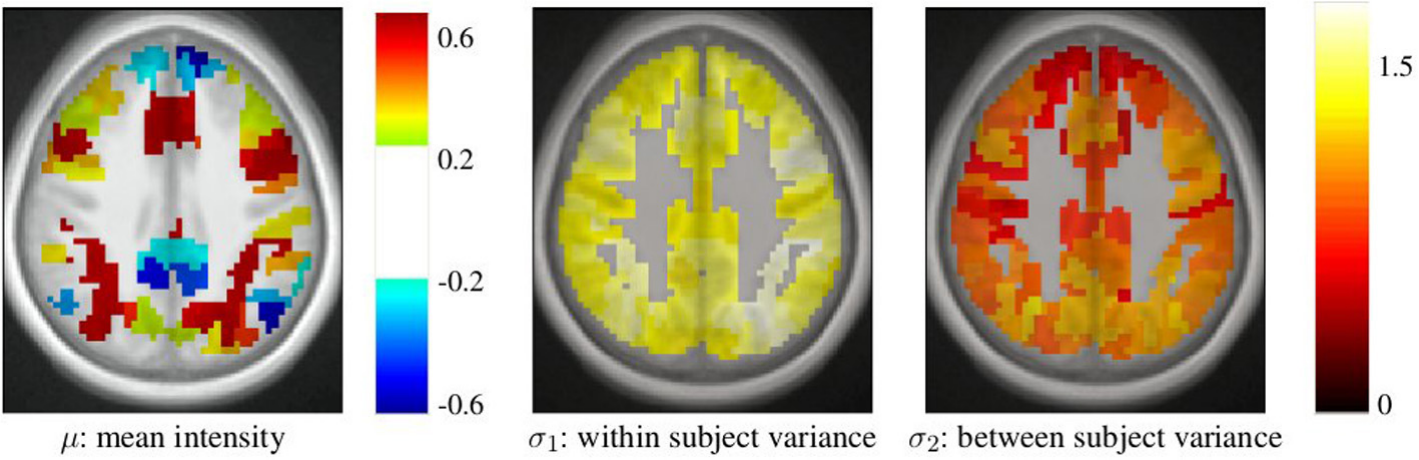
**Example of parameters estimated in a parcellation obtained with Ward’s clustering and *K* = 500 parcels**. They are given in arbitrary units (percent of the baseline fMRI signal, squared for variance estimates). These parameters are those for the *computation-sentence reading* functional contrast. μ: mean intensity, σ_1_: within subject variance, and σ_2_: between subject variance.

**Figure 5:**
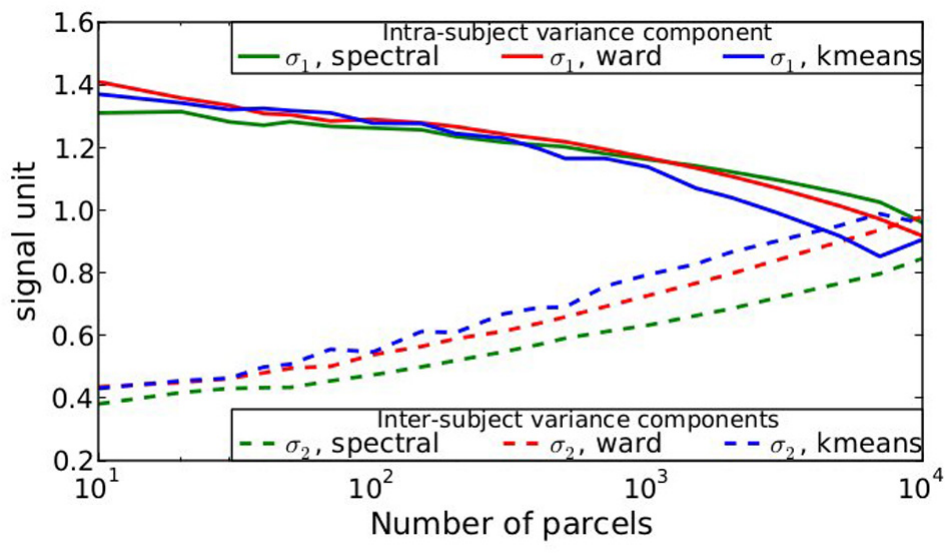
**Dependence on *K* of the variance components from model (Equation 6), averaged across parcels and contrasts: both σ_1_ and σ_2_ parameters show a monotonic behavior: the within subject variance decreases σ_1_ with *K*, while the between-subject variance σ_2_ increases with *K***.

After parcellation, the parameters of the model (Equation 6) are estimated in each parcel, for each functional contrast and can be plotted in the brain volume; see Figure 4. In particular, it can be seen that σ_1_ > σ_2_ uniformly i.e., within-parcel variability dominates across-subject variability when *K* = 500. Moreover, in the case of Ward’s parcellation presented here, the within- and between-subject variance estimated are quite homogeneous across the brain volume. Note, however, that there is a tendency for both to be correlated with the absolute value of the mean signal. Next, we consider how the variance components, averaged across parcels, change with *K* in Figure 5. These values evolve monotonously with *K*: the intra-subject parameter σ_1_ (that measures the cross-voxel variance within a given subject, averaged across parcels) decreases monotonously with *K*, as expected; the inter-subject parameter σ_2_, that characterizes the cross-subject variability of the mean signal within a parcel, increases monotonously. Both parameters come close to equality for large values of *K* (about 5000). These trends are similar across clustering techniques. This actually means that changing the resolution yields a re-allocation of the variance from the intra-subject to the inter-subject component of the mixed-effects model. More specifically, for low values of *K*, the high within-subject variance shadows the between-subject variance, and a very large value of *K* has to be used if one wants to estimate correctly the between-subject variability of the BOLD signal within the parcellation framework.

***4.2.1.1. Comparison with an anatomical atlas***. As a basis for comparison with anatomical atlases, we evaluated the log-likelihood of the data with the most detailed atlas that we could find. We used the Harvard–Oxford atlas both cortical and subcortical http://fsl.fmrib.ox.ac.uk/fsl/fslwiki/Atlases together with the cerebellar atlas (Diedrichsen et al., 2009). The version used was that of FSL 4.1. The regions were systematically divided into left and right hemispheres by taking the sign of the *x* MNI coordinate of the voxels. Using this procedure, we obtained 158 regions. This atlas was resampled at the resolution of the test fMRI data, and the likelihood of the data summed over parcels was evaluated and compared with that of data-driven parcellations with 158 parcels, obtained either from the Localizer dataset itself or from the HCP dataset. Standard deviation of the log-likelihood are obtained by drawing *B* = 30 bootstrap samples. The results are shown in Table 1.

**Table 1:** **Summed log-likelihood of the Localizer data under different spatial models (the higher, the better): brain atlas (left), parcellation on the Localizer dataset (middle), parcellations from the HCP data (right)**.

**Atlas**	**Summed log-likelihood**	**Stdv**
Harvard–Oxford atlas	−6.642 10^7^	1.9 10^5^
Geometric parcellation	−6.589 10^7^	1.9 10^5^
k-means parcellation	−6.463 10^7^	1.8 10^5^
Ward parcellation	−6.513 10^7^	1.9 10^5^
Spectral clustering parcellation	−6.591 10^7^	1.8 10^5^
(HCP) k-means parcellation	−6.710 10^7^	1.9 10^5^
(HCP) Ward parcellation	−6.522 10^7^	1.9 10^5^
(HCP) Spectral clustering parcellation	−6.613 10^7^	1.9 10^5^

The number of parcels used is K = 158 for all methods. The standard deviation is obtained by drawing 30 bootstrap samples.

We show the corresponding results on the HCP dataset in Table 2.

**Table 2:** **Summed log-likelihood of the HCP data under different spatial models (the higher, the better): brain atlas (left), parcellation on the HCP dataset (middle), parcellations from the Localizer data (right)**.

**Atlas**	**Summed log-likelihood**	**Stdv**
Harvard-Oxford atlas	−4.557 10^7^	3.3 10^5^
Geometric parcellation	−4.537 10^7^	3.4 10^5^
k-means parcellation	−4.459 10^7^	3.1 10^5^
Ward parcellation	−4.491 10^7^	3.4 10^5^
Spectral clustering parcellation	−4.543 10^7^	3.3 10^5^
(Localizer) k-means parcellation	−4.529 10^7^	3.3 10^5^
(Localizer) Ward parcellation	−4.530 10^7^	3.3 10^5^
(Localizer) Spectral clustering parcellation	−4.539 10^7^	3.3 10^5^

The number of parcels used is K = 158 for all methods. The standard deviation is obtained by drawing 30 bootstrap samples.

It can be seen that the anatomical atlas achieves the poorest fit: summarizing the fMRI data on the corresponding set of parcels loses a lot of information. Even a purely geometric parcellation performs better, which can be understood given that it tends to create parcels with equal size, hence achieves a more regular sampling of the volume of interest. For *K* = 158 the best performing parcellation on the training set is obtained from k-means, but these parcellations do not generalize well from a dataset to another. Ward’s parcellation on the other hand, performs better than geometric clustering in all configurations. Finally, the bootstrap variability of these results is typically small with respect to between-method difference for the Localizer dataset, ensuring that the differences are significant. This is less so with the HCP dataset, for two reasons: the number of subjects is smaller, and the per-subject SNR seems relatively lower in that dataset (see Barch et al., 2013).

#### 4.2.2. Analysis of the goodness of fit the models (localizer dataset)

**Figure 6:**
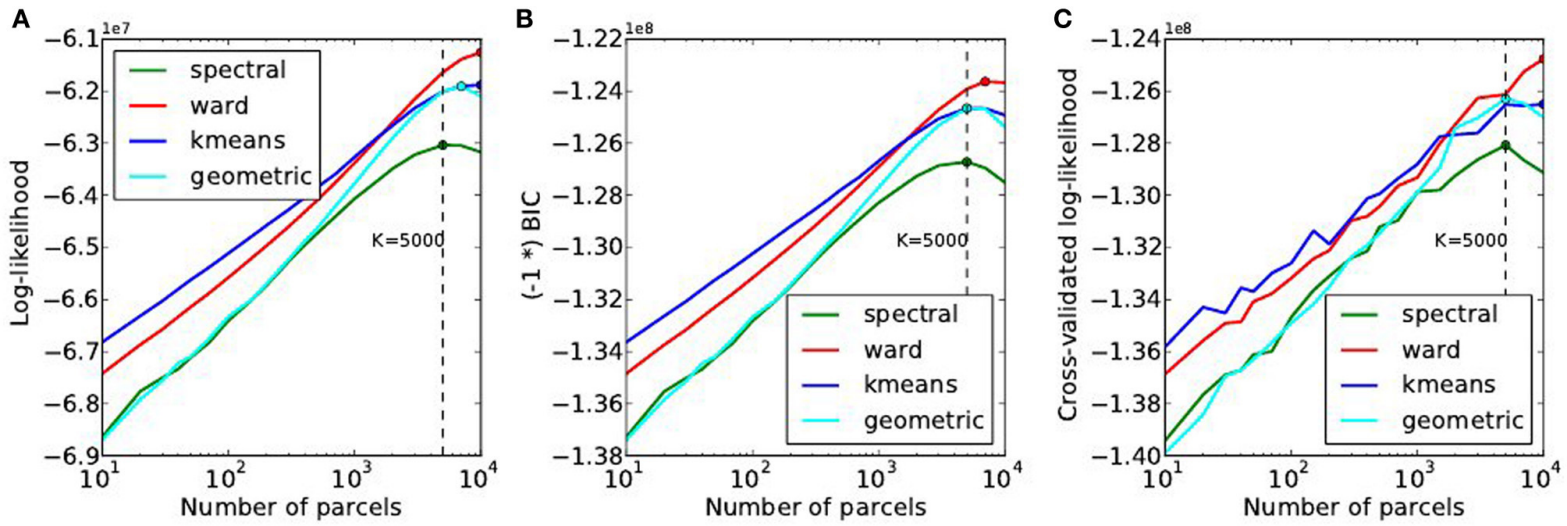
**Distortion metrics. (A)** Accuracy of the model (Equation 6) measured through the summed Log-likelihood across parcels, as a function of the number *K* of clusters. The accuracy is maximized for very high values of *K*. The Bayesian Information Criterion **(B)** with the sign flipped for the sake of visualization- and the cross-validated log-likelihood **(C)**, that can be used to identify the right model show the same behavior as the log-likelihood function.

The goodness of fit of the model is given by the log-likelihood, which can be compared across methods for a fixed value of *K* in Figure 6A. The main observations are:

- For all methods, the curve achieves an optimum value for a very large number of parcels (3000 ≤ *K* ≤ 7000), which is much more that the number typically expected and used in neuroimaging experiments.
- *k-means* and *Ward’s* clustering achieve the lowest distortion—i.e., loss of information from the original signal—with *k-means* performing better for low number of parcels and *Ward’s* clustering performing better for large number of clusters. Spectral clustering is inferior in terms of goodness of fit. It is even lower than a purely geometric parcellation of the brain volume for some values of *K*.
- The achieved log-likelihood is larger on smoothed data than on unsmoothed data, but the behavior is qualitatively similar. In this report, we present only results on unsmoothed data.

Second, we can observe that, unlike in our simulations, BIC and cross-validated log-likelihood (Figures 6B,C) achieve their optimum at the same value of *K* as the data log-likelihood function, thus at very high values (3000 ≤ *K* ≤ 7000).

#### 4.2.3. Accuracy-reproducibility compromise (localizer dataset)

**Figure 7:**
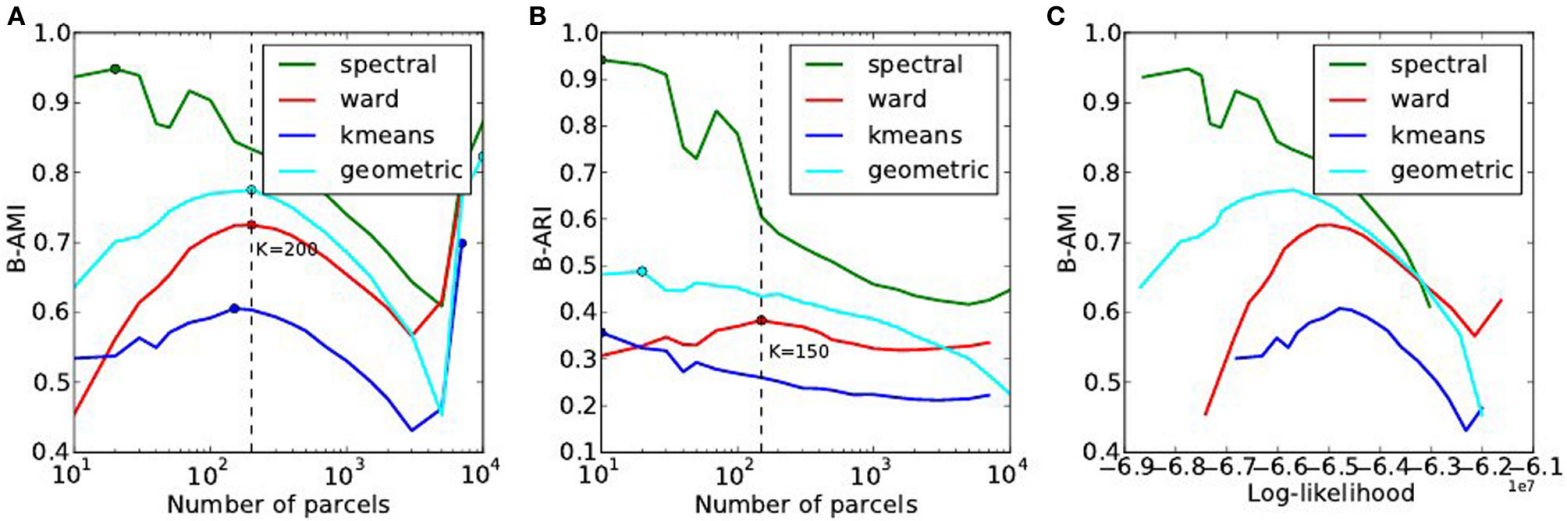
**Analysis of the reproducibility index with respect to the number of parcels (A,B) and with respect to the negative log-likelihood (C)**. For all methods but one, the B-AMI (Bootstrapped Adjusted Mutual information) index **(A)** shows a (local) maximum for about 200 parcels and decreases against for larger numbers, until it increases again for very large number clusters (*K* ≥ 5000). By contrast, B-ARI (Bootstrapped Adjusted Rand Index) **(B)** only displays the local maximum on Ward’s parcellation. If we consider B-AMI against accuracy there is thus a trade-off region, for a number of parcels comprised between 200 and 5000 (decreasing portion of the curves in the reproducibility-accuracy curve), in which each setting represents a different compromise. The two dominant techniques are spectral clustering, that maximizes the reproducibility index, and Ward’s clustering, that yields higher accuracy overall.

The reproducibility of the clustering estimated by bootstrapping the data can be studied as a function of the number of clusters, or as a function of the likelihood. Both representations are presented in Figure 7.

The reproducibility index displays a clear optimum value at *K* ≃ 200 parcels. For larger values, the reproducibility index decreases slowly, but increases again for very large number of parcels *K* > 4000. This late increase can readily be interpreted as an artifact due to the fact that we are now observing a very large number of very small clusters, and that the reproducibility indexes are not well suited in this case. It is also true that very small clusters tend to represent the spatial neighboring system, and thus this high reproducibility is not very informative on the functional features carried by the data.

The spectral clustering outperforms the other alternatives regarding reproducibility, which means that it is able to capture some stable features in the input data, although the overall representation is suboptimal in terms of accuracy. Regarding the sensitivity/reproducibility compromise (see Figure 7, right), the spectral method is dominant in the low accuracy/high reproducibility region, while Ward’s method dominates in the high accuracy/low reproducibility region.

### 4.3. Model selection results on the HCP dataset

**Figure 8:**
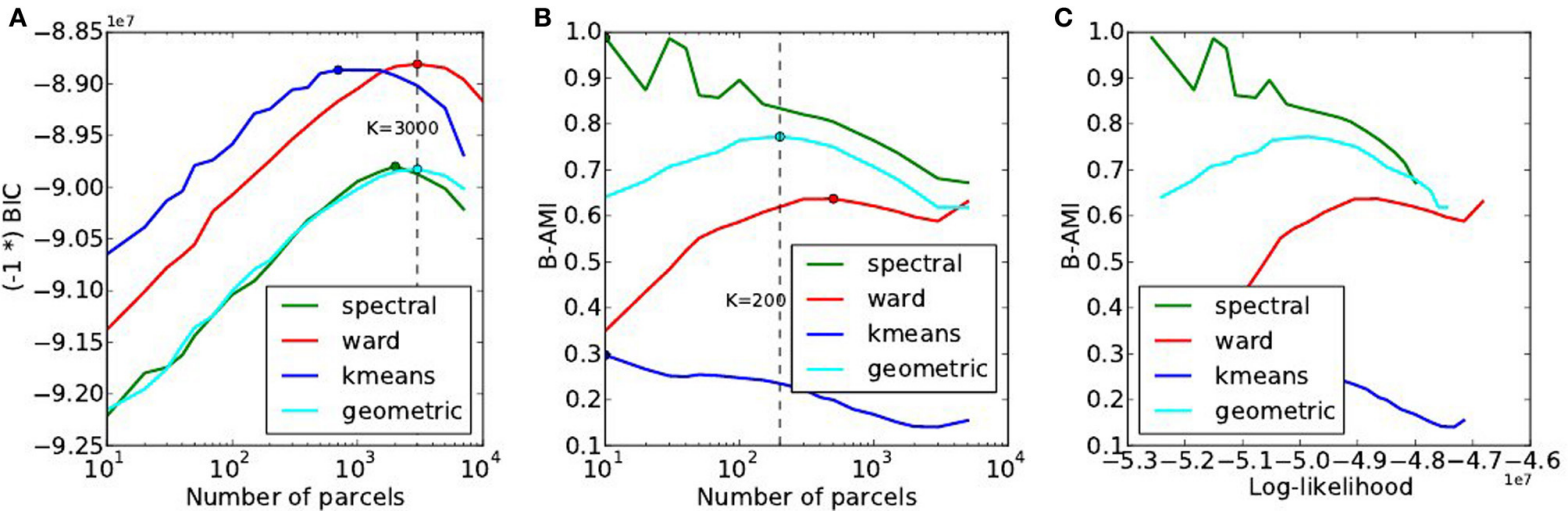
**Results of the of the model selection experiments on the HCP dataset. (A)** Accuracy-based selection through the BIC score, **(B)** reproducibility-based selection through Bootstrapped Adjusted Mutual Information, **(C)** ensuing sensitivity/reproducibility curve.

A summary of the results obtained by doing the same experiments on the HCP dataset is provided in Figure 8. In spite of weak changes of the optimal values K^*^ ≤ 3000 for accuracy, K^*^ ∈ [200, 500] for reproducibility, this dataset reproduces exactly the trends observed with the Localizer dataset: Ward’s method outperforms the others in terms of accuracy and for high *K* values, spectral clustering yields a poor fit and a high reproducibility, k-means a good fit, especially for small *K*, yet very low reproducibility.

## 5. Discussion

Our experiments benchmark three methods to derive brain parcellations from functional data, using three model selection criteria and two reproducibility measures. Though not exhaustive, these experiments are very informative on the general behavior, the domain of optimality of the methods, and the issues that limit the power of such approaches in neuroimaging data analysis.

### 5.1. Guidelines for functional parcellation extraction

#### 5.1.1. Which criterion to use for methods comparison?

To frame the problem, it is necessary to choose the criterion used to guide model selection. Note that this is an important yet difficult aspect of any unsupervised statistical learning procedure. We studied two different characteristics of functional parcellation that are critical to their usage in brain mapping: how well they capture the functional signal and how reproducible they are under perturbations of the data. To measure the goodness of fit of the functional signal, it is important to distinguish within-subject variance from across-subject variance, as only the first kind of variance is minimized when the number of parcels increases. Our probabilistic model offers a natural goodness of fit criterion, the log-likelihood; by penalizing it (BIC criterion) or by using cross-validation, it is possible to obtain a sound model selection. Our simulations show that cross-validation almost systematically outperforms BIC, but we did not notice systematic differences in the real dataset. The other important aspect of a brain description is its stability, and we also investigated other criteria that the selected number of clusters based on the consistency of parcellations. This approach behaved similarly as the others on synthetic data, but provided a much more conservative selection on real data (*K* ~ 200–500 parcels according to the dataset and method). The fact that reproducibility and accuracy yield different decisions for model selection is well known, and has been illustrated in neuroimaging by LaConte et al. (2003); this effect is tightly related to the classical bias /variance in statistics.

#### 5.1.2. Which algorithm to prefer?

Regarding the clustering algorithms themselves, our general finding is that Ward’s algorithm should be preferred, unless a small number of parcels is required. Indeed, spatially-constrained Ward’s clustering outperforms the other techniques in the large *K* regime (say, *K* ≥ 500) in terms of goodness of fit, while having fair results in terms of reproducibility. With respect to k-means, it offers the additional advantage of providing spatially connected parcels. In theory, k-means algorithm should do better in terms of accuracy, but the optimization problem solved by k-means is hard (non-convex) and thus bound to sub-optimal solutions; as a consequence the greedy approach in Ward’s algorithm outperforms it. Moreover, k-means based parcellations tend to fit data idiosyncrasies and thus do not generalize well across datasets, as shown in Tables 1, 2. We observed that mixture models would behave similarly to k-means, since k-means is in fact a constrained Gaussian mixture model with hard assignments. In a side experiment, we observed that Gaussian mixture models perform consistently better than k-means, but the difference is tiny and comes with high computational cost.

Spectral clustering is not a powerful approach to outline structures in the data. A simple geometric clustering procedure is as good, and sometimes better in terms of accuracy. The reason is that spectral clustering is efficient with high SNR data when clusters are easily discriminated, which is not the case with functional neuroimaging data, where it mostly outlines geometrical structures. Similar observations were made in Craddock et al. (2012). Note, however, that spectral clustering is even more stable than geometric clustering, meaning that it captures some structure of the input data.

#### 5.1.3. How many parcels?

It should first be emphasized that choosing the number of parcels in our model is not exactly the question of deciding how many functional regions can be found in the brain, but how many piecewise constant models can actually be fit to some fMRI data reliably. The distinction is important, because some regions, for instance V1, will contain internal functional gradients, such as those related to retinotopy, orientation sensitivity and ocular dominance. In theory, the function specificity could therefore be resolved at the level of columns in these regions, but this does not mean that larger structures do not exist. The conclusions that we draw here are bound to the data that we have used and generalization to different modalities or contrasts (resting-state fMRI, anatomical connectivity) is not guaranteed.

The goodness of fit-related criteria yields high numbers (up to *K* = 5000 for Ward’s clustering, slightly less for the others, but this may simply reflect a lack of sensitivity of these approaches in the large *K* regime, in which Ward’s clustering fits the data better), simply indicating that functional activations cannot easily be represented as piecewise constant models: whether this is an intrinsic feature of brain function or an impact of cross-subject spatial mismatch or pre-processing artifacts remains an open question. In the future, the use of brain registration algorithms based on functional data (Sabuncu et al., 2010) may significantly affect model selection.

The reproducibility criterion, on the other hand, peaked at *K* ~ 200, meaning that there is probably a relevant level of description with such a resolution. Thus, when parcellations are used to obtain a model of brain organization that seeks to characterize individually each parcel, a conservative choice *K* ~ 200–500 should be preferred for the sake of reproducibility. Note that *K* = 200 is a lower bound on the right dimensionality, i.e., models with a resolution lower than 200 regions are not flexible enough to represent functional signals without introducing severe distortions. In particular, anatomical atlases that propose a decomposition into about 100 regions, are not sufficient to summarize functional signals, some of the resulting ROIs being very large.

Yet, the problem of optimizing the number of parcels remains open and should be addressed in a data-driven fashion.

### 5.2. Challenges and further work

#### 5.2.1. The difficulty of model selection on noisy data

It is important to remember that discovering functionally homogeneous structures is a hard problem, given that the SNR of the data is low, and that even visual inspection would most often be insufficient to define a relevant parcellation. Besides this issue, neuroimaging data come with additional difficulties: the data are smooth, which could be accounted for but is not in model (Equation 6). The other difficulty is that the spatial jitter brought by imperfect spatial normalization, and poor matching between functional organization and sulco-gyral anatomy across subjects makes this an ill-posed problem, since regions with homogeneous functional characteristics may be slightly displaced across individuals, which invalidates the model hypotheses. Both smoothing and jitter break the hypotheses of BIC, which yields poor model selection. Cross-validation and reproducibility are more resilient to this effect.

#### 5.2.2. Limitations of this experiment

Our experiments are based on two datasets, with a pre-defined set of contrasts. We have been able to check that using any of the contrasts or all of them yields qualitatively similar results (data not shown). The power of the experiment is that it is based on a relatively large number of subjects (67–128), so that one can at least conjecture that the between-subject variability observed in functional neuroimaging is correctly sampled. Note that the Localizer data come from two different scanners, resulting in an un-modeled latent factor. We observed, however, that our conclusions were unaltered when performed on a subset of subjects coming from the same scanner (data not shown).

The model that we use has several limitations:

- The parcellation itself is fixed across subjects. While a relaxation to individual dataset has been proposed in Thirion et al. (2006), such a procedure loses some of the properties of clustering, and make model selection much harder.
- Our model (Equation 6) does not account for spatial effects in the within-parcel covariance, which would probably make it more robust to data smoothness and possibly to cross-subject spatial jitter, but the computational price to pay for these models is high.
- It assumes that the true activation signal is piecewise constant. A smooth interpolation scheme between parcels might make it more powerful, hence reducing the requirement of large *K* values. Again, this would increase the complexity of the model fitting.

#### 5.2.3. Suggestions for population-level fMRI modeling

One of the observations made in this study is that the problem of the spatial jitter across subjects remains the main limitation that needs to be overcome in order to learn appropriate population-level atlases. This should be addressed using procedures such as those presented in Sabuncu et al. (2010) and Robinson et al. (2013). Other improvements of the model concern the possibility of using not a single parcellation, but several different parcellations and to aggregate the results (i.e., the significant effects across subjects) by marginalizing the parcellation as a hidden variable of parametric models (Varoquaux et al., 2012; Da Mota et al., 2013). Besides, different parcellation schemes could use different values for *K*. In particular, Ward’s algorithm is a hierarchical algorithm, that can actually be used to estimate multi-scale representations of brain activity (see e.g., Michel et al., 2012; Orban et al., 2014). Specifically Orban et al. (2014) suggest that the hierarchical organization of nested clusterings obtained from hemodyamic response function would be stable in the population, hinting at an intrinsic feature of brain organization. This is an additional asset of this procedure that has not been considered in this work but could be used in future applications of brain parcellations.

## Funding

The authors acknowledge support from the ANR grant BrainPedia ANR- 2010-JCJC-1408-01 and the Human Brain Project.

### Conflict of interest statement

The authors declare that the research was conducted in the absence of any commercial or financial relationships that could be construed as a potential conflict of interest.

## Footnotes

^1^Often, a small sphere will be drawn around this position to average signals locally.

^2^A variant of spectral clustering replaces this k-means step by learning a rotation to discretize the representation (Yu and Shi, 2003). We used this approach, that outperforms k-means, in the case where the number of desired clusters *K* is smaller than the subspace dimension *m* (see next).
